# Version 4 of the CRU TS monthly high-resolution gridded multivariate climate dataset

**DOI:** 10.1038/s41597-020-0453-3

**Published:** 2020-04-03

**Authors:** Ian Harris, Timothy J. Osborn, Phil Jones, David Lister

**Affiliations:** 1 National Centre for Atmospheric Science, Centre for Ocean and Atmospheric Sciences, School of Environmental Sciences, University of East Anglia, Norwich, NR4 7TJ UK; 2 Climatic Research Unit, School of Environmental Sciences, University of East Anglia, Norwich, UK

**Keywords:** Climate and Earth system modelling, Atmospheric dynamics

## Abstract

CRU TS (Climatic Research Unit gridded Time Series) is a widely used climate dataset on a 0.5° latitude by 0.5° longitude grid over all land domains of the world except Antarctica. It is derived by the interpolation of monthly climate anomalies from extensive networks of weather station observations. Here we describe the construction of a major new version, CRU TS v4. It is updated to span 1901–2018 by the inclusion of additional station observations, and it will be updated annually. The interpolation process has been changed to use angular-distance weighting (ADW), and the production of secondary variables has been revised to better suit this approach. This implementation of ADW provides improved traceability between each gridded value and the input observations, and allows more informative diagnostics that dataset users can utilise to assess how dataset quality might vary geographically.

## Abstract (summary)

**Table:** 

Measurement(s)	temperature • volume of hydrological precipitation • vapour pressure • wet days • cloud cover
Technology Type(s)	digital curation
Factor Type(s)	date of observation • location of observation
Sample Characteristic - Environment	climate system
Sample Characteristic - Location	Asia • Africa • Europe • Australia • North America • South America

Machine-accessible metadata file describing the reported data: 10.6084/m9.figshare.11980500

## Background & Summary

The CRU TS (Climatic Research Unit gridded Time Series) dataset provides a high-resolution, monthly grid of land-based (excluding Antarctica) observations going back to 1901 and consists of ten observed and derived variables (Table 1 introduces their acronyms and other relevant information). There are no missing values in the defined domain. Individual station series are anomalised using their 1961–1990 observations, then gridded to a 0.5° regular grid using angular distance weighting (ADW); the resulting anomaly grids are converted to actuals (actual values, ie, not anomalies) for publication using the CRU CL v1.0 climatologies^1^.

**Table 1:** CRU TS variables, showing codes, units, correlation decay distances (CDDs) and precursors.

Variables	Code	Units	CDD (km)	Precursors
**Variables - primary**				
Mean 2 m temperature	TMP	degrees Celsius	1200	None
Diurnal 2 m temperature range	DTR	degrees Celsius	750	TMN, TMX databases
Precipitation rate	PRE	mm/month	450	None
**Variables - secondary**				
Vapour pressure	VAP	hPa	1000	TMP, DTR
Wet days (Notes 1, 2)	WET	days	450	PRE
Cloud cover	CLD	percentage	600	DTR
**Variables - derived**				
Frost days (Note 3)	FRS	days per month	750	TMN
Minimum 2 m temperature (Note 4)	TMN	degrees Celsius	1200	TMP, DTR
Maximum 2 m temperature (Note 4)	TMX	degrees Celsius	1200	TMP, DTR
Potential evapo-transpiration (Note 5)	PET	mm/day	n/a	TMP, TMX, TMN, VAP, CLD

Note 1: A wet day is one receiving ≥0.1 mm precipitation.

Note 2: Used in diverse areas, including evaluation of satellite observations^32^ and evaluation of potential evapotranspiration equations^36^.

Note 3: Also used in many areas, including dendroclimatology^37^ and health^38^.

Note 4: Used to calculate scPDSI for monitoring drought^39^, and in areas including regional agronomic production^40^ and river basin vegetation^41^.

Note 5: minimum and maximum temperatures are the monthly means of the individual daily minimum and maximum temperatures; they are not the overall minimum or maximum temperature recorded in each month.

CRU TS was first published in 2000^2^, using ADW (angular-distance weighting) to interpolate anomalies of monthly observations onto a 0.5° grid over land surfaces (excluding Antarctica) for seven variables (Table 2). The selection of ADW as the interpolation method was made after extensive evaluation of alternatives [2, section 2b]. Updates in 2004^3^, 2005^4^ and yearly from 2006 to present^5^ increased the variable count to ten (Table 2), and switched to triangulation (utilising IDL functions including TRIGRID and TRIANGULATE) to effect the interpolation and perform much of the synthetic variable work (remaining code was in Fortran). Synthesised observations were interpolated onto a coarser grid (2.5°, regular) and used to ‘plug gaps’ in the observed coverage. An extensive account of these processes may be found in references^2^ and^5^, particularly with respect to filling in gaps in coverage.

**Table 2:** CRU TS major versions, showing included variables (‘**X**’).

Version	TMP	DTR	PRE	VAP	WET	CLD	TMN	TMX	FRS	PET
1.0	X	X	X	X	X	X			X	
2.0	X	X	X	X		X				
2.1	X	X	X	X	X	X	X	X	X	
3.0	X	X	X	X	X	X	X	X	X	
3.1	X	X	X	X	X	X	X	X	X	X
4.0	X	X	X	X	X	X	X	X	X	X

Since the first release in 2000, CRU TS has been used widely by many classes of user, in diverse research areas and applications. These include those with localised weather- and climate-dependent models (for example, river catchment^6^, agronomic^7^), those calibrating paleoclimate reconstructions^8,9^, those analysing climate variability^10^, and those needing bias correction for global^11^ and regional climate models^12^ and reanalyses^13^. Away from the sphere of climate research, users include the civil engineering^14^, financial^15^ and insurance^16^ sectors.

This version seeks to implement a more streamlined process, with ADW improving interpolation efficiency and accuracy, and delivering a full suite of metadata to facilitate nuanced interpretation of the gridded values and full traceability where necessary for quality control. This has been enabled by the move to a fully bespoke process, implemented in Fortran and described in the ‘Methods’ and ‘Data Records’ sections of this paper. The choice to return to ADW was driven by the need for improved traceability; it is justified by this, and supported by the comparison of interpolation methods reported in^2^.

**Fig. 1:**
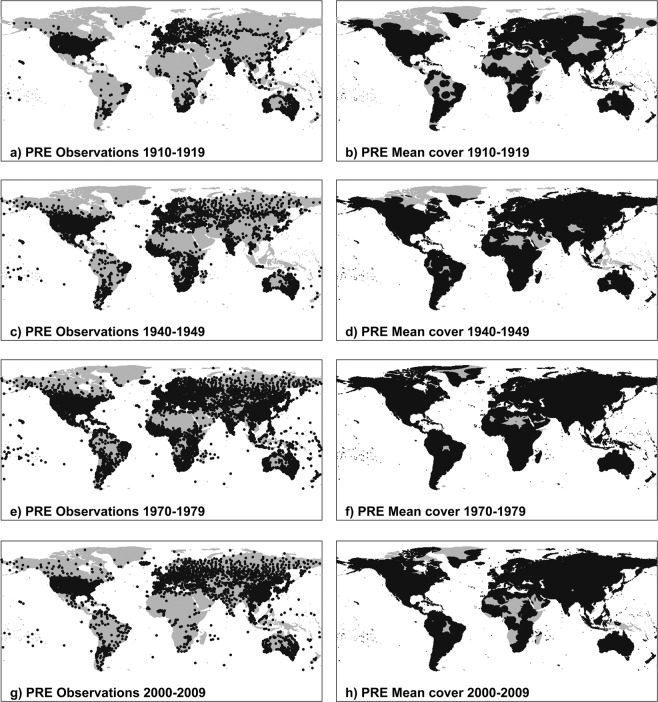
Station coverage for PRE (total precipitation). Decades included are 1910–1919 (**a**,**b**), 1940–49 (**c**,**d**), 1970–79 (**e**,**f**) and 2000–09 (**g**,**h**), showing station locations (left column) and resulting cover (right column). Additional cover from the background climatology is not shown. The CDD for PRE is 450 km. Stations appear if they contribute at least 75% of observations in the decade; grid cell cover is shown where a gridcell has interpolated data for at least 75% of time steps in the decade. For this reason, discontinuities may be observed between each decadal pair.

**Fig. 2:**
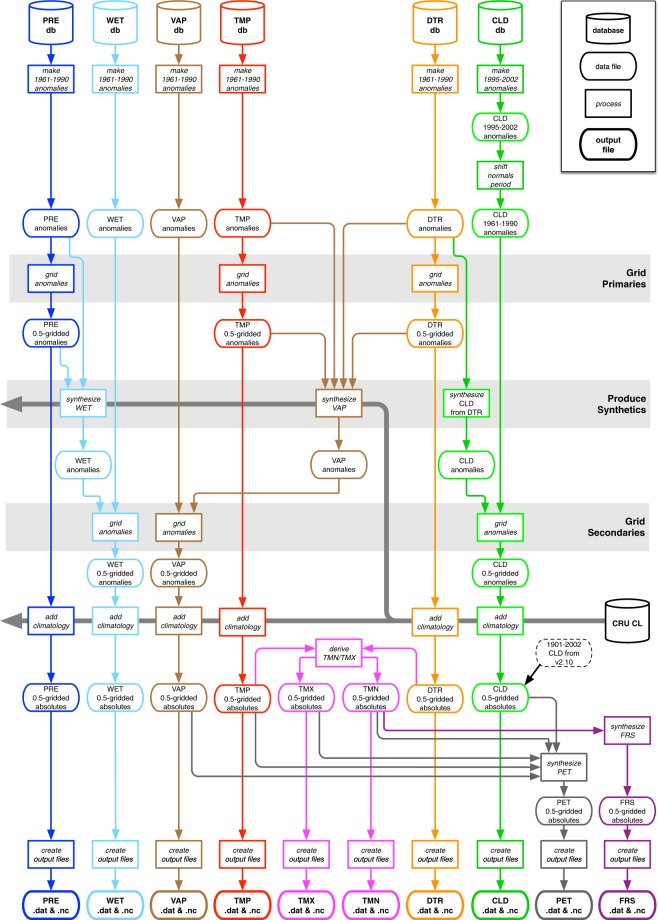
CRU TS production process. Colours show construction routes for each variable (see Table 1 for details of the variables).

Monthly land station observations for seven variables (Mean, Minimum and Maximum Temperatures, Precipitation, Vapour Pressure, Wet Days and Cloud Cover) are updated regularly from several principal monthly sources: CLIMAT messages, exchanged internationally between WMO (World Meteorological Organisation) countries, obtained as quality-controlled files via the UK Met Office; MCDW (Monthly Climatic Data for the World) summaries, obtained from the US National Oceanographic and Atmospheric Administration (NOAA) via its National Climate Data Centre (NCDC); and updates of minimum and maximum temperatures for Australia, obtained from the Bureau of Meteorology (BoM). In addition, ad-hoc collections of stations are incorporated (after quality control checks including location, correspondence to existing holdings, and outlier checking). These observations serve to provide six ‘databases’ of monthly values (Diurnal Temperature Range being calculated from Minimum and Maximum Temperatures). Coverage for selected variables at selected dates is shown for precipitation in Fig. 1 and for temperature, DTR and vapour pressure in the three figures of Supplementary File 1, and discussed further in the ‘Meteorological station database updating’ subsection of ‘Methods’. Figure 2 shows the overall process by which these observations, along with various static repositories, are used to derive each version of the CRU TS data set. Further variables are derived from these, including Potential Evapotranspiration (PET), which is required by many users in the agricultural and hydrological sectors.

Because of the overriding objective to present complete coverage of land surfaces (excluding Antarctica) from 1901 onwards, CRU TS is not *necessarily* an appropriate tool for assessing or monitoring global and regional climate change trends. Nevertheless, with care taken to identify and avoid trend artefacts caused by changing data coverage or data inhomogeneities, then CRU TS *can* be used for global and regional trend analysis. The first issue is that unlike, for example, CRUTEM, regions uninformed by observations are not left missing but instead are replaced by the published climatology^1^. This has the advantage of being a known entity, rather than an estimate, but has the unavoidable side effect of decreasing variance. Additionally, the numbers and locations of stations contributing to any grid cell will change over time. Both effects can potentially give rise to trend artefacts. This is a particular problem with high-resolution grids, if individual grid cells or small groups of grid cells are analysed without checking to see if they contain any observation stations at all, or whether they are interpolated from distant stations during one part of the record and from close stations during another period. However, the metadata provided with the CRU TS version 4 dataset enables users to understand the level of support behind each grid cell and time step, permitting informed detection of trends or masking of areas so that analysis of trends can focus on well-observed regions. Temperature, in particular, has been shown to be resilient to the problems described above: this is in part due to its long correlation decay distance (CDD) of 1200 km (Supplementary File 1). Precipitation, with its much shorter CDD of 450 km, has reduced and more time-dependent coverage (Fig. 1), and so subject to these problems unless the data are masked prior to analysis. The second issue is that no extra homogenization is performed on the observations, so artefacts could be present where the originators have not already homogenized their data. Comparisons with other observation-based datasets at a global scale (GPCC^17^, UDEL^18^, CRUTEM^19^, and regional or third-party exercises^20–22^) demonstrate the robustness of the dataset at large spatial scales. Assessment of the grids is discussed further in the ‘Technical Validation’ section of this paper.

## Methods

### Meteorological station database updating

The process to update the databases with observations, and to derive the DTR database, is unchanged and is described in^5^. Holdings of observations vary by variable, with spatial and temporal concerns affecting cover. In Fig. 1, and in Supplementary File 1 (three figures), the left column shows station locations in different decades: valid observations with at least 75% in a decade (ie, 90 or more monthly observations) are required for inclusion here. The right columns show the resultant gridded cover, taking into account the correlation decay distances (CDDs) of the variables: again, interpolated data for a minimum of 75% of the decade (90 or more values) are needed for a grid cell to be shaded. CDDs for CRU TS variables were established^2^. Figure 1 shows PRE station cover for 1910–19, 1940–49, 1970–79 and 2000–09. There are far more PRE stations than for any other variable, but its CDD is the lowest (450 km), and so regions with sparse support have patchy coverage. The PRE database has been evaluated against other precipitation station collections in^23^. In Supplementary File 1, TMP station cover (p.2) shows that in the early 20th century, even the high CDD of TMP cannot deliver full land cover: central-west Africa being the most obvious region that will default to the climatology. DTR cover (p.3) has far patchier cover than TMP, owing to its shorter CDD (750 km) and lower station numbers. The final figure in Supplementary File 1, VAP station cover (p.4) demonstrates the difference between the cover provided by VAP observations, and that introduced with the addition of synthetic VAP: for this reason, only two decades (1940–49 and 1970–79) are shown. The comparisons between b) and d), and between f) and h), show how essential synthetic variables are to achieving much greater land cover. Note that the synthetic VAP, as it is derived from TMP and DTR, inherits the lower of their CDDs (750 km). Cover is therefore reduced from that of TMP.

### Anomalies

The first stage of the process is to convert each station series into anomalies. The mean used to construct the anomalies is based on the period 1961–1990, and a minimum of 75% of observations must be present in this period (23 months or more) for each of the 12 months to be processed. Outlying values exceeding a threshold (±3 standard deviations, SD, for TMP; +4 SD for PRE) are omitted. This outlier-threshold check for TMP is more stringent than that for CRUTEM4.6, which uses a ±5 SD outlier check^24^. In total the ±3 SD check removes 8.6% of the TMP values. For regions where anomalies were exceptional (>3 SD) this can potentially remove correct values. Of the 8.6%, 8.4% and 0.2% were respectively negative and positive extremes. Although the outlier checks are strong, this does not adversely affect the later, broad comparisons discussed in the ‘Technical validation’ section. While the process to construct anomalies is algorithmically unchanged from the previous version^5^, additional elements now construct a lookup table which, for each anomalised station, lists all land cells for the destination 0.5° grid that are within the correlation decay distance (CDD) for the variable in question. This improves the computational performance of the later interpolation process.

### Production of primary variables: TMP, DTR, PRE

Primary variables have no synthetic component. Station observations are anomalised using each station’s 1961–1990 normals (monthly averages). PRE is converted to percentage anomalies, so the lowest possible value would be −100, meaning no rain; and a percentage anomaly of 0 indicates equivalence with the 1961–1990 mean. Monthly anomaly fields are then interpolated onto the 0.5° × 0.5° target land grid using ADW. Land grid cells where no observation reaches are set to 0 (representing the climatology in anomaly space). Finally, the CRU CL published climatologies are used to convert the gridded anomalies to actuals.

### Secondary variables

Secondary variables differ from primary variables in that they have fewer direct observations available. We therefore supplement these by estimating synthetic values from the primary variables. The synthetic estimates are obtained using empirical relationships with the primary variables that are unchanged from those described in^5^. What has changed in CRU TS4 is that the synthetic estimates are now calculated from the primary variable *station* observations rather than from the primary variable *gridded* values. Two advantages of this change are that (1) it is more transparent which stations have contributed to the gridded values (those with observations of the secondary variable and those with observations of the primary variable(s) needed to obtain the synthetic estimates); and (2) the interpolation of the synthetic estimates can now use the CDD of the secondary variable in deciding the distance weighting. Previously, some synthetic estimates were derived from gridded primary variables that had themselves been interpolated using the CDD of the primary variable (hence less transparent, and information from further afield than the secondary variable’s CDD would have been used). One result of this is that the coverage (the regions where the variable is not simply filled in by its climatological values) of the secondary variables is less complete than previously. However, this reduction in coverage arises from removing potentially low quality estimates that were previously made from too-distant observations.

#### Synthetic VAP production

**Fig. 3:**
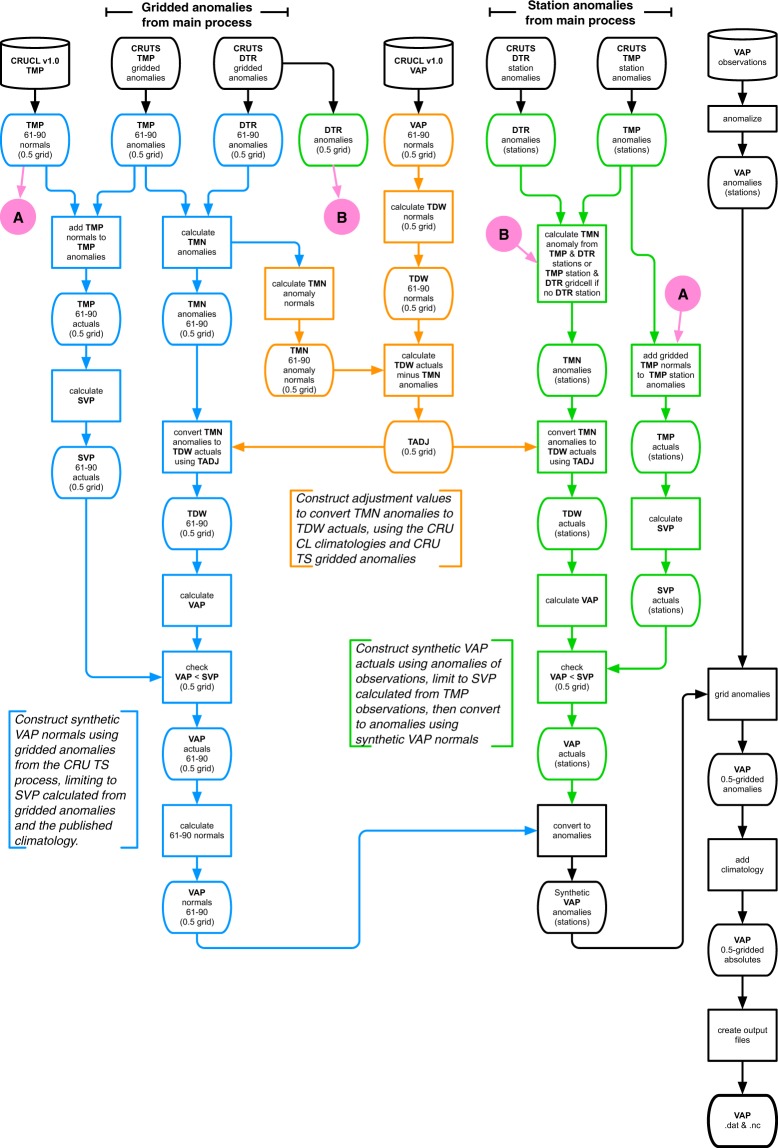
VAP (vapour pressure) production process. Colours show subprocesses. TDW is dewpoint temperature; SVP is saturation vapour pressure.

Synthetic VAP observations are generated from TMP and DTR station anomalies (or from TMP station anomalies and gridded DTR anomalies where the station data does not include TMN and TMX), as well as the published CRU climatologies for TMP and VAP^1^. While the process broadly follows that described in^5^, synthetic anomalies are now produced at a station level, rather than as gridded data, because this better suits the interpolation process as explained above. The VAP process is shown in Fig. 3, and the impact of the inclusion of synthetic VAP on the final gridded coverage is illustrated in Supplementary File 1 (p4).

#### Synthetic WET production

**Fig. 4:**
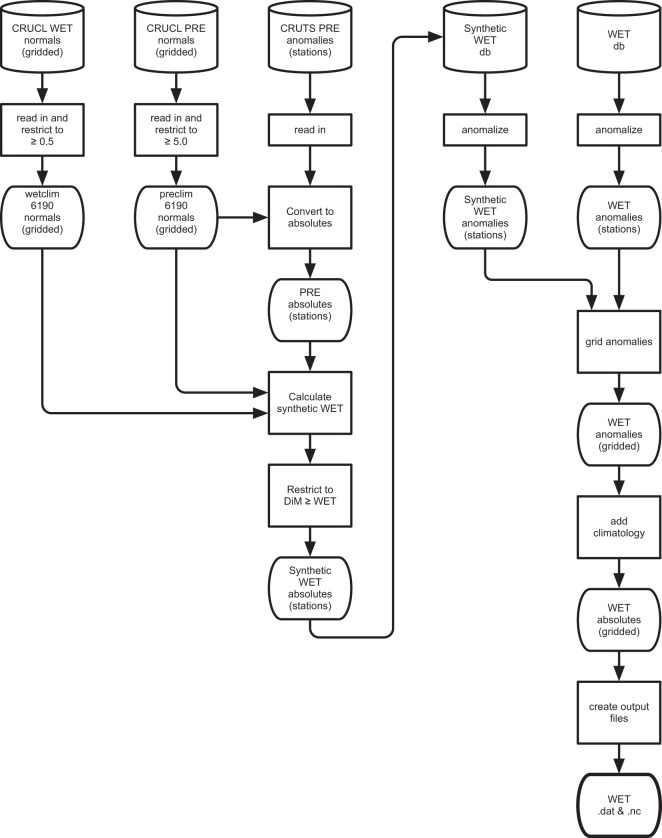
WET (wet-days) production process. DiM is the number of days in the month.

The WET variable represents counts of wet days defined as having ≥0.1 mm of precipitation (section 2.4.1 of^5^). Figure 4 shows the process by which synthetic WET values are incorporated into production of the WET product. The empirical algorithm that synthesizes WET uses PRE observations, together with normals (the CRU CL 1961–1990 climatologies^1^) for PRE and WET. Therefore, the PRE anomalies at a station level are converted to absolute values using the PRE normal from the enclosing gridcells, and then used in the synthesis. The absolute synthetic WET values produced go to create a synthetic WET database; this is then anomalised in the same way as the observed WET database, and both sets of anomalies are passed to the interpolation algorithm. Some users use WET, and as rain day counts are part of the monthly messages we access, they are straightforward to add to the databases.

#### Synthetic CLD production

The process to generate synthetic cloud cover observations from DTR observations is as described in^5^, save that the synthetic station-based values are not gridded separately, but are fed into the main gridding process alongside the CLD observation anomalies.

### Interpolation

#### General approach

**Fig. 5:**
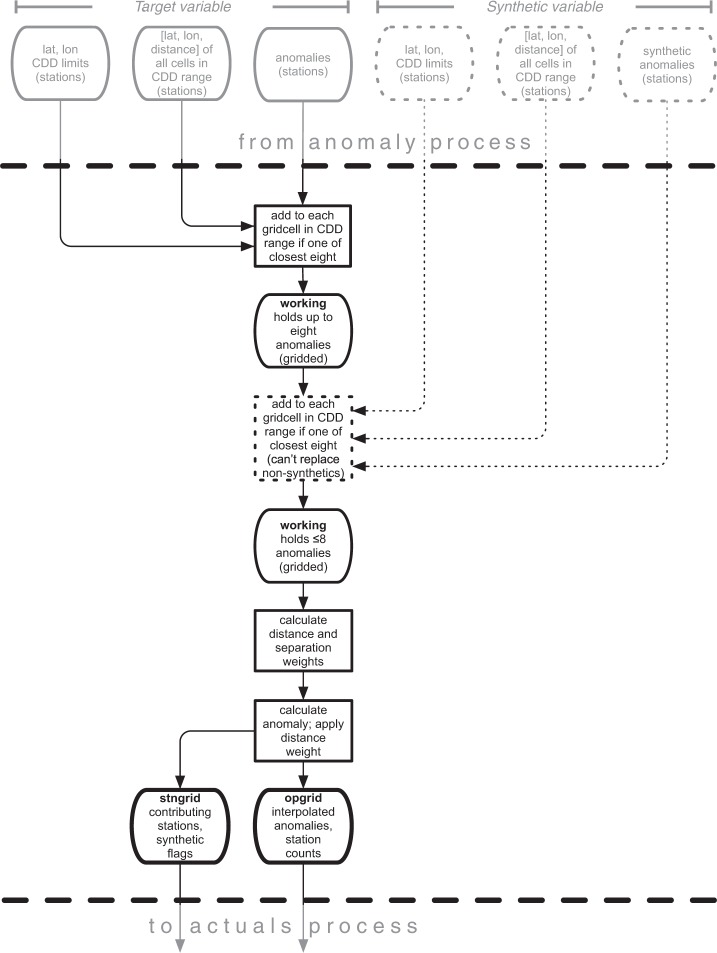
Interpolation process. Showing the optional addition of synthetic anomalies (dotted lines). Names in bold refer to variables in the relevant program. CDD is the correlation decay distance.

The interpolation process implements angular-distance weighting (ADW) and is shown in Fig. 5. The station influence lookup tables produced as part of the anomaly process (described in the ‘Anomalies’ subsection of ‘Methods’) are used to allocate station anomalies to an array of gridcells that, for each monthly time step and cell, stores the nearest eight or fewer anomalies lying within the relevant CDD. Once the observed anomalies have been allocated, and if a secondary variable is being processed, synthetic anomalies are then allocated in the same way. However, they are excluded if within 25 km of either an observed anomaly, another synthetic anomaly, or the centre of the target cell; and if they lie within a 45° subtended angle of an observed anomaly. Additionally, they cannot replace an observed anomaly: the maximum of eight anomalies applies throughout. Once all allocations have been made, distance and (angular) separation weights are calculated (section 2b of^2^), and used to obtain an interpolated anomaly value for each gridcell. Any land cells without allocated anomalies are set to zero, representing the climatology in anomaly space. Elevation is not specifically included in the interpolation; it is introduced via the climatologies when the gridded anomalies are converted to absolute values (‘Production of absolutes’ in ‘Data records’). Results of a cross-validation exercise to quantify the accuracy of the ADW interpolation scheme are reported in the ‘Technical validation’ section.

#### Improvements to weighting for v4.02 and later versions

The approach to distance-weighting adopted for version 4 was taken from^2^, a decay function of the form ${\left({e}^{-d/CDD}\right)}^{m}$, where *d* is the distance of the station, *CDD* is the correlation decay distance of the variable, and *m* = 4 (a value arrived at after extensive sensitivity testing reported in^2^). However, the function was only used as part of the ADW process, when weighting more than one station to achieve an interpolated value. This resulted in unrealistic artefacts in the interpolated field. To address this, there was a need for the interpolated anomaly - or, for a single station, its anomaly - to be damped using distance-weighting as well, and a cross-validation exercise was conducted. This involved the reconstruction of every observed anomaly from every station in the process, provided at least one other station was available to interpolate from. These reconstructions were made for two basic decay functions, the original:

1 $$damping\,factor={\left({e}^{-d/CDD}\right)}^{m}$$

and a sine-based function with a slower decay at closer distances:

2 $$damping\,factor=1-\sin {\left(rad\left(d/CDD\right)\right)}^{n}$$

In both cases, the power *m* or *n* ranged in integer steps from 1 to 8. The interpolation process applied the selected function at all stages: the decay of a lone station anomaly with distance, the relative distance weighting in the ADW calculation, and the decay of the ADW-derived anomaly with distance. Errors were calculated as mean absolute error (MAE) for various regions, including latitude bands and 5° × 5° gridcells, as well as global values. In all cases, either Eq. (1) with *m* = 8 or Eq.(2) with *n* = 1 gave the smallest errors as a global picture: PRE was served equally by both, while TMP was served better by Eq. (2). However, this sine curve does not allow a gradual decay at close distances, resulting in unrealistic artifacts as before. Further calculations showed that increasing the power in the sine function introduced little extra error, and a value of *n* = 4 was selected as a compromise between the need for accuracy in the gridcells and the need to reduce or eliminate unrealistic artifacts in the field to provide a continuous surface.

### Derived variables (TMN, TMX, FRS and PET)

TMN and TMX are derived arithmetically from the gridded absolute values of TMP and DTR, as described in^5^. FRS is derived entirely synthetically, using an empirically determined function of the gridded absolute TMN variable. Potential Evapotranspiration (PET) is calculated using the Penman-Monteith formula^25^ explained in^26^ (p1071–1072). For this we use the CRU TS gridded values of mean temperature, vapour pressure, cloud cover and static (temporally invariant except for the annual cycle) 1961–90 average wind field values (further described in^5^).

### Consistency between variables

**Fig. 6:**
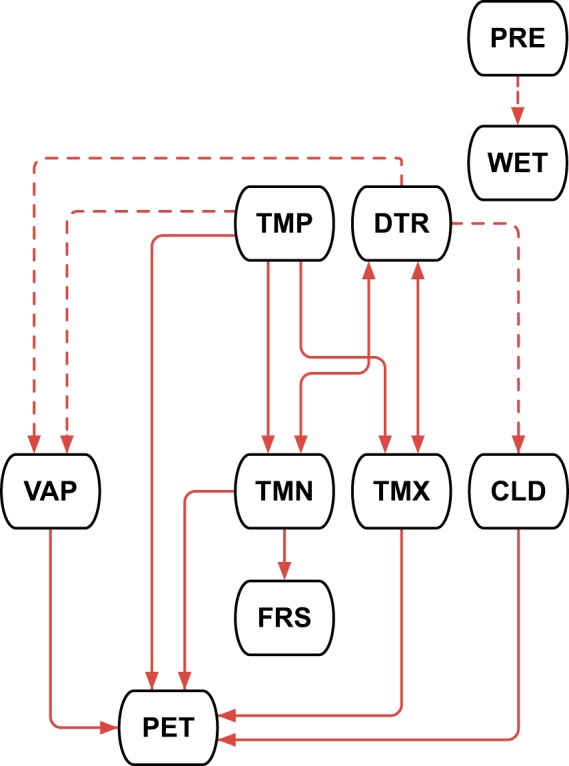
Consistency between variables. The arrowheads indicate the direction of data ensuring consistency; note that TMP and TMX are derived from DTR (and TMP), but DTR is derived from them earlier (as observations), so these lines are bidirectional. Dashed lines indicate partial consistency, where the synthetic element of the recipient variable will be consistent with the donor variable, but the observed element cannot be said to be so.

One of the benefits of a multivariate dataset is the opportunity to present, at a point in space and time, a set of variable values that are (to an extent) internally consistent. This explains much of the design of the variable production process: TMN and TMX are consistent with TMP and DTR because they are derived from them (DTR having been previously derived from TMN and TMX observations); VAP is consistent with the temperature variables inasmuch as synthetic VAP is derived from them; similarly, the synthetic parts of WET and CLD are consistent with, respectively, PRE and DTR; and FRS and PET are entirely consistent with other variables, being wholly derived from them. Figure 6 shows the consistency relationships.

### Homogeneity

As described in^5^, and in the ‘Production of primary variables’ subsection of ‘Methods’, CRU TS is not specifically homogeneous. Some National Meteorological Agencies (NMAs) homogenize their station observations, either before release or at a later stage (requiring a re-release). Therefore, many CRU TS observations have been homogenized (and also quality controlled) within each country. However, performing additional homogenization on the CRU TS databases would be complicated and not completely possible because of elements of the process, such as partly synthetic variables and the use of published climatologies. Sparse data coverage in some regions, or for some variables, is a particular limitation for applying neighbour-based homogeneity tests, as noted by^4^, where a degree of homogenization was implemented. The multivariate nature of CRU TS means that homogeneities identified in, for example, mean temperature data, are likely to influence other variables as well.

**Fig. 7:**
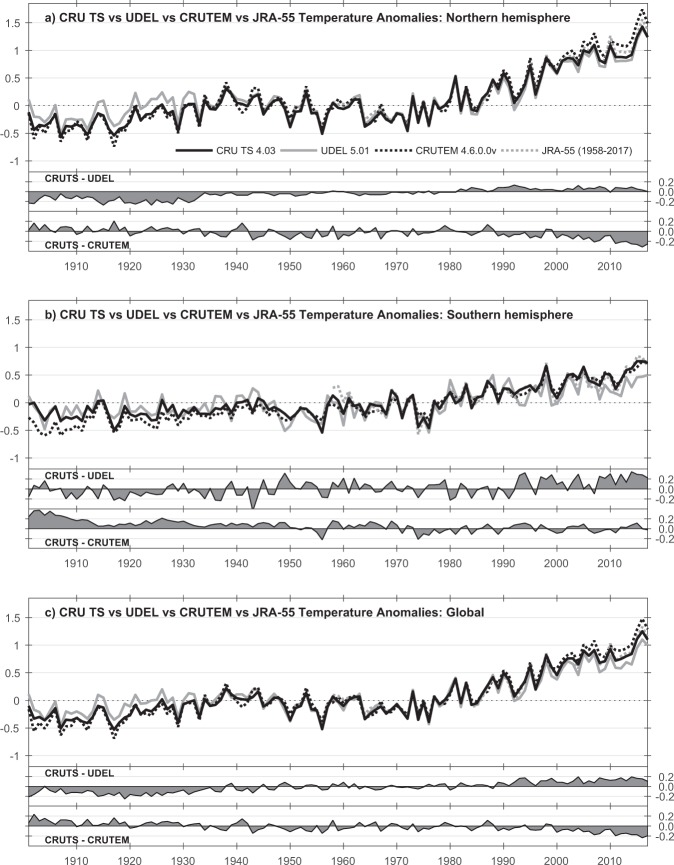
Comparisons of hemispheric and global annual temperature means. From CRU TS v4.03, UDEL v5.01, CRUTEM v4.6.0.0 (variance-adjusted), and JMA JRA-55 reanalysis. All values are anomalies with respect to 1961–1990. Separate difference plots also shown for UDEL and CRUTEM.

Comparisons with other datasets can be used to identify any large inhomogeneities that might be present in CRU TS v4. For example, partial homogeneity assessment and correction was undertaken for an earlier version (v2.1) of CRU TS^4^ and at large spatial scales and for most country averages there is close agreement between CRU TS versions with and without this additional homogenization. Other, single-variable datasets perform various homogeneity assessments on their observations, though even here there are difficulties because of reporting delays^17^. The CRUTEM4.6 temperature dataset incorporates homogeneity as a result of previous work and work by originating bodies [24, section 2.2]. CRU TS v4 TMP is compared with CRUTEM4.6 in the ‘Technical validation’ section and Fig. 7. These various inter-dataset comparisons do not indicate that there any large inhomogeneities present in the CRU TS v4 dataset, unless they are also present in the comparison datasets despite these other data being subject to further homogeneity checks.

## Data Records

### External data records

The CRU TS v4.03 dataset^27^ comprises ten variables of high-resolution global land surface gridded absolute values. The data are available in two formats: NetCDF, and space-separated ASCII text. This ensures maximum availability for the diverse users of the dataset. The files are available in decadal blocks, as well as full-length, for the same reason.

The gridded data, excepting PET, are made available alongside metadata indicating the level of station support enjoyed by each datum; this varies between 0 (no cover, climatology inserted, see Interpolation above), and eight (the maximum station count for interpolation). For primary (TMP, DTR, PRE) and secondary (VAP, WET, CLD) variables, the counts produced by their interpolation are used. For derived (TMN, TMX) variables, and for FRS, the DTR counts are used. Because PET is calculated from multiple variables using a Penman-Monteith formula^25^, no meaningful station count can be produced. The station count metadata are included in the NetCDF files as a second variable (‘stn’), and are published separately as ASCII text files.

An interface is also provided by a file in Keyhole Markup Language (KML) and an accompanying suite of images and datafiles. This is a standard of the Open Geospatial Consortium (https://www.opengeospatial.org/standards/kml) and allows the data set to be accessed in Earth browsers such as Google Earth (https://earth.google.com/). This Google Earth interface is currently available for the TMP and PRE data, allowing access to individual grid-cell series as well as station observations in an intuitive, hierarchical structure.

CRU TS is available from the Centre for Environmental Data Analysis (CEDA: http://data.ceda.ac.uk//badc/cru/data/cru_ts/), and from the CRU website: https://crudata.uea.ac.uk/cru/data/hrg/ (which also hosts the Google Earth interface structures).

### Internal data records

The CRU TS process is realised through a collection of Fortran-77 programs that are called from a master program. This arrangement has provided compartmentalisation and flexibility as the process has evolved. This section will address the data files that allow communication between the programs, organised by the program that produces the data files. All files are ASCII text, with space-separated fields, unless otherwise stated.

#### Anomaly production

The anomaly program produces monthly data files, listing the station anomalies for that month. Station metadata is included. Additionally, two files needed for the interpolation process are produced: a list of stations giving in grid terms the North, South, East and West bounds of their influence (based on the CDD of that variable); and a list of stations giving the co-ordinates and distances of all gridcells within that influence. Files produced by the anomaly process are used by the interpolation process. Additionally, anomalies for primary variables are used by the processes synthesizing VAP, WET and CLD.

#### Synthetic VAP production

The synthetic VAP program produces monthly data files in ASCII text, listing the synthetic anomalies for that month. Station metadata is included. Because the process can make use of gridded DTR anomalies if there is no match for a TMP station, the metadata can take one of two forms: either a TMP station, or both TMP and DTR stations. These files are used by the interpolation process.

#### Synthetic WET production

The synthetic WET program produces monthly data files in ASCII text, listing the station absolutes for that month. Station metadata is included. The format is identical to the station record format used for observations, and the files are read by the anomaly process.

#### Synthetic CLD production

The synthetic CLD program produces monthly data files in ASCII text, listing the station anomalies for that month. Station metadata is included. The format is compatible with the anomaly files produced by the anomaly process, and these files are used by the interpolation process.

#### Interpolation

The interpolation program produces monthly gridded data files in ASCII text, comprising the gridded anomalies for that month. These files are used by the absolutes process. A separate monthly file identifies, for each datum, the number of stations that contributed to the interpolation, these files are used by the output process. A further monthly file identifies, for each datum, the stations used and whether they were observations or synthetic. This latter file is not currently used by any process, but it exists to provide full traceability when required.

#### Production of absolutes

The absolutes program reads the gridded anomaly files for primary and secondary variables from the interpolation process, and converts them to absolutes using the appropriate CRU CL v1.0 climatology. It produces monthly gridded files, which are used by the output process as well as in the derivation of TMN and TMX, and the calculation of FRS and PET.

#### Derivation of TMN and TMX

The program that derives TMN and TMX does so by reading the monthly gridded files of absolutes for TMP and DTR, produced by the absolute process. It produces monthly files of TMN and TMX in the same format, which are read by the output process. TMN is calculated as TMP − 0.5*DTR, and TMX as TMP + 0.5*DTR.

#### Synthetic FRS production

The synthetic FRS program reads monthly gridded TMN absolutes produced by the absolute process, and produces monthly files of FRS in the same format. These are read by the output process.

#### Synthetic PET production

The synthetic PET program reads monthly gridded TMP, TMN, TMX, VAP and CLD absolutes produced by the absolute process, and produces monthly files of PET in the same format. These are read by the output process.

#### Output process

The output process reads the gridded absolute files produced by the absolute process, and the station count files produced by the interpolation process. It produces the final output files described in the ‘External data records’ subsection of ‘Data records’.

## Technical Validation

### Quality control of input data

Source observations are often homogenized by national meteorological agencies before dissemination. Addition of new observations includes basic range checking, where the observations are from a trusted service, and interactive operator-controlled addition in other cases.

No achievable level of quality control can guarantee to exclude all errant data from a large dataset, because of the myriad ways in which the data may be evaluated and the elusive definition of ‘errant’. The disparate users of CRU TS subject the data to many kinds of statistical processing, and on occasion this can reveal potential issues for further exploration and perhaps correction.

The process of anomaly production includes screening of exceedences; these are defined as values exceeding three standard deviations (based on the full length of the station series), extended to four standard (positive) deviations for precipitation.

### Comparisons between versions and with alternative datasets

When CRU TS v4 was introduced, CRU TS v3 continued to be produced in parallel, to allow users to investigate how the move would affect their work. So v4.00 was released alongside v3.24(0.01), v4.01 alongside v3.25, and v4.02 alongside v3.26 (the latter being the final version of CRU TS v3). Comparison plots, using country mean annual series, are available for each pair of releases: v4.00 against v3.24.01 (https://crudata.uea.ac.uk/cru/data/hrg/cru_ts_4.00/comparisons.32401.vs.400); v4.00 against v2.10 (https://crudata.uea.ac.uk/cru/data/hrg/cru_ts_4.00/comparisons.210.vs.400); v4.01 against v3.25 (https://crudata.uea.ac.uk/cru/data/hrg/cru_ts_3.25/observation.v3.25.v4.01); and v4.02 against v3.26 (https://crudata.uea.ac.uk/cru/data/hrg/cru_ts_4.02/observation.v3.26.v4.02). These comparisons illustrate the impact of the modified interpolation process introduced with v4; as both methods use the same station databases, only the processes are different.

At a global scale, there are very few datasets available for comparison. For TMP, The University of Delaware’s 0.5° dataset^18^ has been used, along with CRU’s CRUTEM dataset^19^ and the reanalysis dataset JRA-55 from JMA^28^. For PRE, DWD’s GPCC^17^ was chosen for its high observation count. TMP comparisons (Fig. 7) show good high-frequency agreement of CRU TS with CRUTEM4.6 (correlation coefficient, *r* = 0.99 globally), UDEL (*r* = 0.97 globally) and JRA-55 (*r* = 0.99 globally, 1958–2017 only). However, the long-term trends in global and hemispheric land temperature are notably stronger in CRUTEM4.6 than in UDEL: the CRU TS trend lies between them but closer to CRUTEM4.6. This is clear in the difference plot (‘CRUTS-UDEL’), with CRU TS being warmer than UDEL in the early Twentieth Century, and cooler more recently. Comparisons between CRUTEM and other global temperature datasets (as reported, e.g., by^29^) support the reliability of the long-term CRUTEM trend). It should be noted that CRUTEM is not a spatially interpolated dataset; this may explain some differences.

**Fig. 8:**
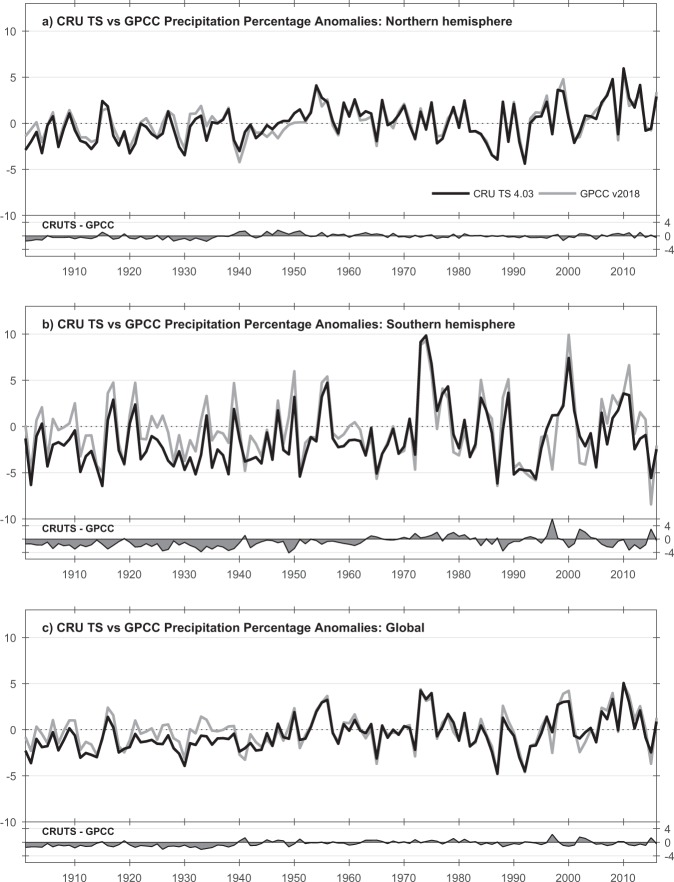
Comparisons of hemispheric and global annual precipitation means. From CRU TS v4.03 and GPCC v2018. All values are percentage anomalies with respect to 1961–1990. Separate difference plots also shown.

Figure 8 shows the comparisons of PRE with GPCC for global- and hemispheric-mean land precipitation. The high-frequency *r* for Global is 0.92, though CRU TS is drier in the early Twentieth Century, perhaps due to having lower observation counts and reduced coverage. The difference is largest in the Southern Hemisphere, while the Northern Hemisphere series agree more closely (annual anomalies correlate at 0.94).

In general, comparison with reanalysis products is not appropriate as a way to validate observation-based datasets. Reanalyses are forecast models constrained by some observed variables. Precipitation, for example, is not usually assimilated. There are many examples of gridded observations being used to ‘bias correct’ reanalyses, a selection that used CRU TS are described in^30,31^, and^13^. CRU TS is also used as independent assessment of other datasets, such as satellite-derived data for recent decades^32^, highlighting the continuing need for a dataset based only on *in-situ* direct observations.

### Cross-validation of the interpolated anomalies

**Fig. 9:**
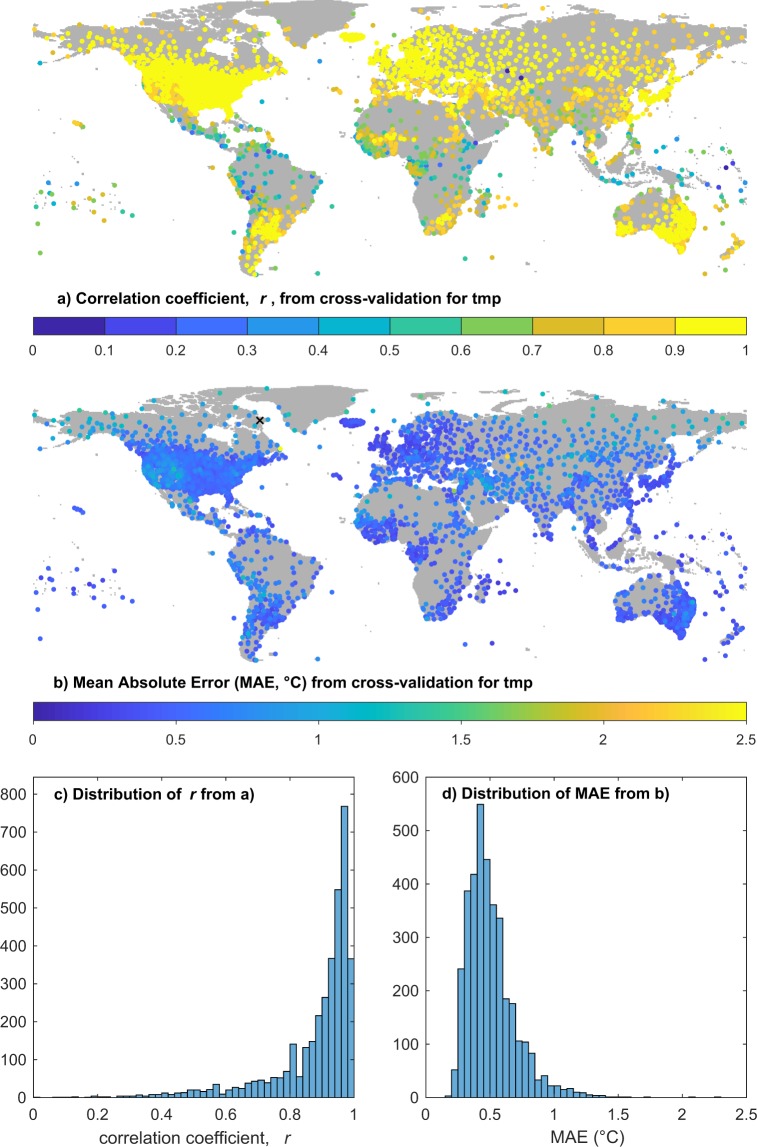
Results of cross-validation for mean temperature (TMP). (**a**) Shows the location and correlation coefficient (r) of each station estimated from nearby interpolants; (**b**) as for (**a**), but showing the mean absolute error (MAE); (**c**,**d**) show the distributions of r and MAE respectively. In order to improve the mapping of colours, the largest MAE value, 4.27 °C, has been replaced with a black cross (at Pangnirtung, on Baffin Island, 66.15°N, 65.72°W).

**Fig. 10:**
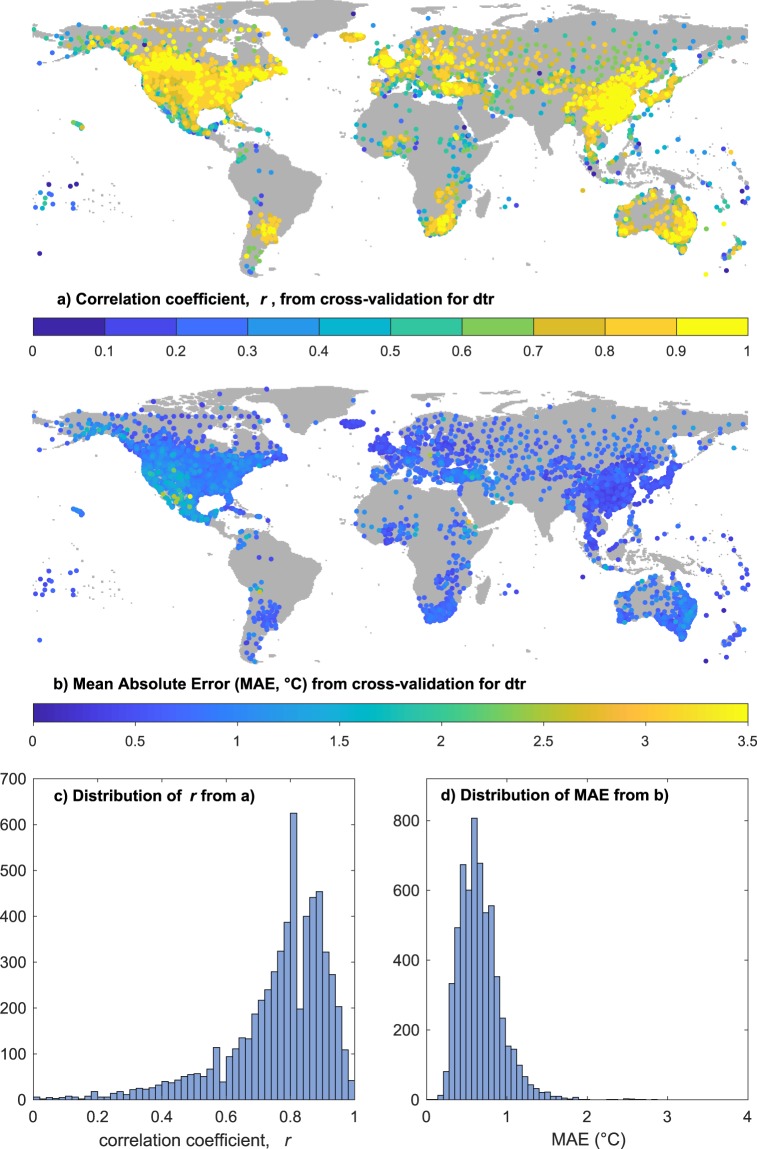
Results of cross-validation for diurnal temperature range (DTR). (**a**) Shows the location and correlation coefficient (r) of each station estimated from nearby interpolants; (**b**) as for (**a**), but showing the mean absolute error (MAE); (**c**,**d**) show the distributions of r and MAE respectively.

**Fig. 11:**
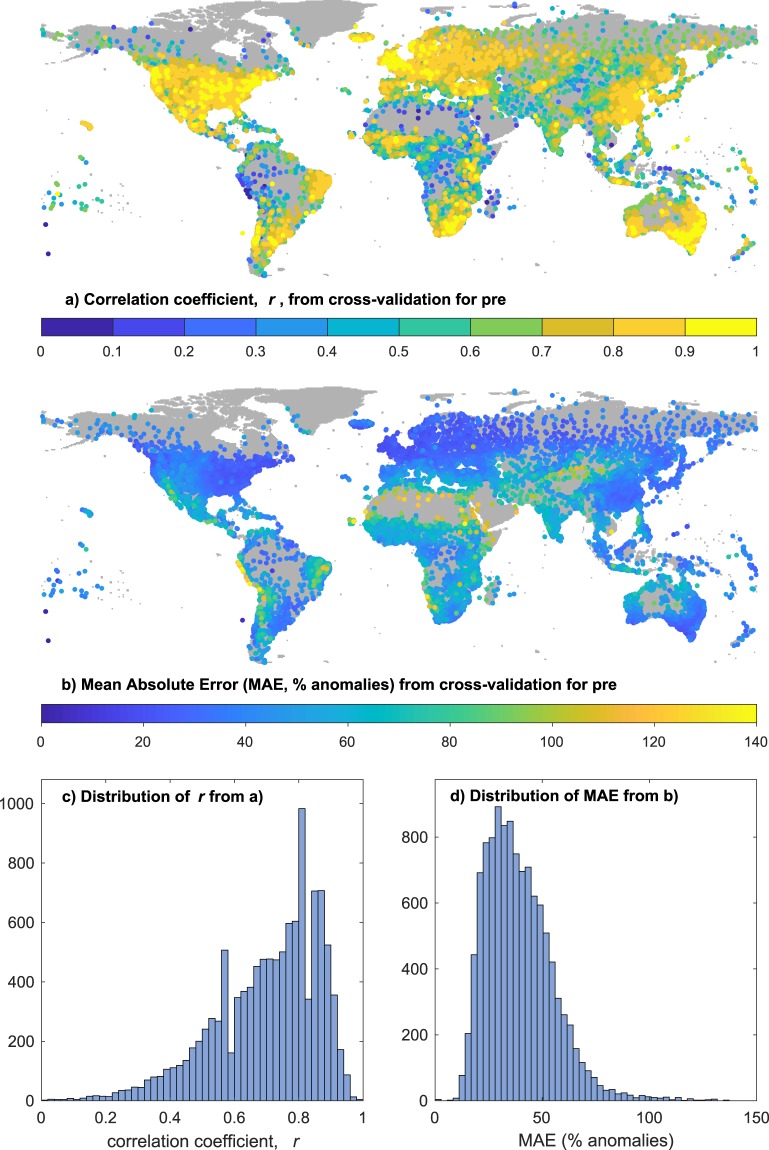
Results of cross-validation for total precipitation (PRE). (**a**) Shows the location and correlation coefficient (r) of each station estimated from nearby interpolants; (**b**) as for (**a**), but showing the mean absolute error (MAE); (**c**,**d**) show the distributions of r and MAE respectively.

A separate suite of skill testing programs use station cross validation^33^ to assess the skill of the interpolation algorithm and to provide a quantitative guide to the expected accuracy of the individual interpolated values. Cross-validation could not have been performed before the move to ADW and is one of the motivations for changing to ADW. Figure 9 shows spatial maps of correlation coefficients (*r*) (a) and (MAE) (b) for TMP stations, with the respective distributions in (c) and (d). Figure 10 has the same format, showing DTR results, and Fig. 11 displays results for PRE. All three figures include distribution graphs for r and MAE: these should be consulted for a global overview of performance. Note that PRE anomalies are percentage differences from the mean rather than in mm units. For all three variables, the defined minimum series length for comparison was 20 months. In practice, 95% of lengths were >=236 months and 99% >=47 months for DTR, higher for TMP and PRE, with minimum lengths of 23 for TMP and PRE, 22 for DTR.

The majority of interpolated monthly temperature anomalies are highly correlated (in 95% of cases, r is >=0.56) with their withheld validation data and mean absolute errors mostly lie between 0.25 and 0.75 °C (MAE is <=0.87 in 95% of cases). Correlations are particularly strong in the densely observed mid-latitudes, and as expected they are weaker where the observation network is sparser and interpolation distances are larger. The monthly DTR anomalies have larger interpolation errors (95% of MAE values are <=0.87 °C) and weaker correlation coefficients, (though 95% of correlations are >=0.41). The pattern of cross-validation outcomes for DTR again shows the most reliable values are in regions with dense observations, e.g. N America, parts of Europe, China and Japan, and well-observed regions of S America, southern Africa and Australia. There is some indication of weaker correlations in coastal regions for the DTR cross validation, but the size of the interpolation errors (MAE) shows a different pattern with a region of raised errors in the SW USA and Mexico and very small errors in Europe and China.

The cross-validation for the interpolated monthly precipitation anomalies shows a broader range of outcomes, consistent with the shorted CDD for this variable, but still overwhelmingly dominated by positive cross-validation correlations (95% are >=0.38). The mode of the distribution of MAE lies just below a 30% relative error, with 95% of MAE <=66.81%. Most of the large relative errors for PRE are in very dry regions, such as the edges of the Sahara and other deserts, and will likely be small in absolute terms. The cross-validation gives correlations above 0.8 for the regions with dense networks. It is more common to find correlation around 0.5 or lower in the regions with sparse data, though it is likely (due to cancellation of the random component of errors) that correlations for seasonal, annual and decadal mean values would be greater than for the monthly values shown here.

Many users have made their own attempts at validation of CRU TS, usually for particular variables (TMP, PRE, DTR). These range from comparison with other, regional data sources (eg.^20,21,34^), to global intercomparisons (eg.^22,35^), using either *in situ*, satellite-based or reanalysis-based data sources. These independent evaluations, of which there are many others, are expected to continue for CRU TS v4. The comparisons with established global datasets (Figs. 10 and 11) described above also serve to underline the validity of key CRU TS variables.

## Usage Notes

The NetCDF-formatted output files of CRU TS data may be read with any NetCDF tools; they are CF-1.4 compliant. Files for all variables except PET contain two data variables, the named one, (i.e., ‘tmp’), and a station count (‘stn’) giving the number of stations used to build each datum. These two variables have identical dimensions. The ASCII text-formatted output files, where the ‘stn’ data are in accompanying files, may be read programmatically. The ‘stn’ data may be used to quantify uncertainty, most particularly by excluding cells with a 0 (zero) count, as these will have been set to the default climatology. Other sources of uncertainty, particularly those associated with the observations themselves, cannot be quantified in the same way: representativeness varies from variable to variable, and we are dealing with monthly means (or totals) rather than daily or sub-daily measurements. The cross-validation exercises summarised in the previous section can give some confidence in the interpolation process itself, but as with all such metadata, it is for the user to decide on boundaries to uncertainty for their particular application.

## Supplementary information

Supplementary File 1

## Data Availability

Code archives for CRU TS releases are available on the CRU website, accompanying each release. The automatic archiving of code for each release was introduced recently, so archives are not available for releases prior to v4.03.
