# Supplementary material for: Version 4 of the CRU TS monthly high-resolution gridded multivariate climate dataset
Source: Sci Data. 2020 Apr 3;7:109. doi: 10.1038/s41597-020-0453-3 (PMC7125108; doi:10.1038/s41597-020-0453-3)
Supplement: Supplementary file 1 [file 41597_2020_453_MOESM1_ESM.pdf]

Version 4 of the CRU TS monthly high-resolution gridded multivariate climate dataset

Supplementary File 1: Contents

Title	Page
Station locations and coverage for TMP (mean temperature)	2
Station locations and coverage for DTR (diurnal temperature range)	3
Station locations and coverage for VAP (mean vapour pressure)	4

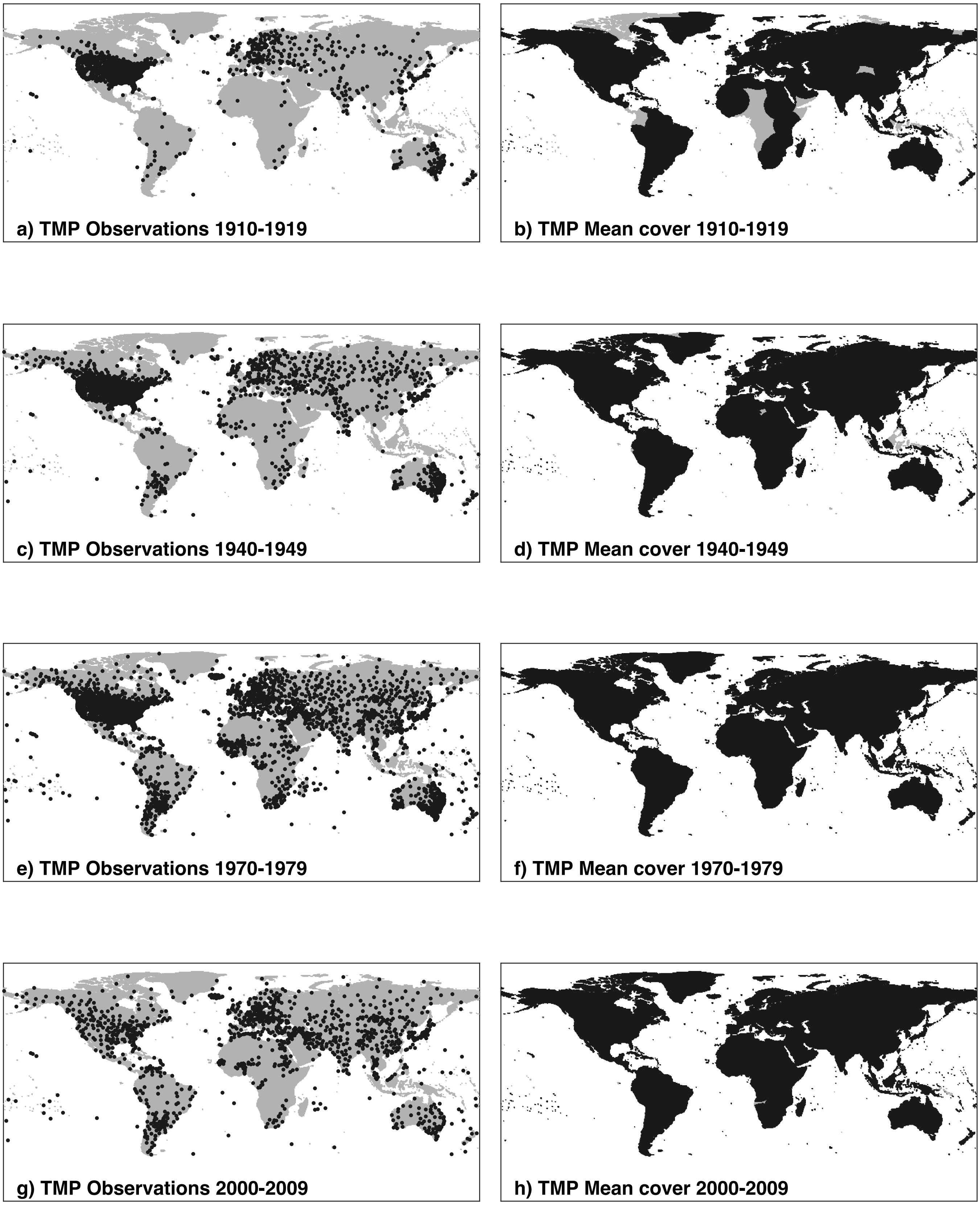

Station locations and coverage for TMP (mean temperature) for four decades

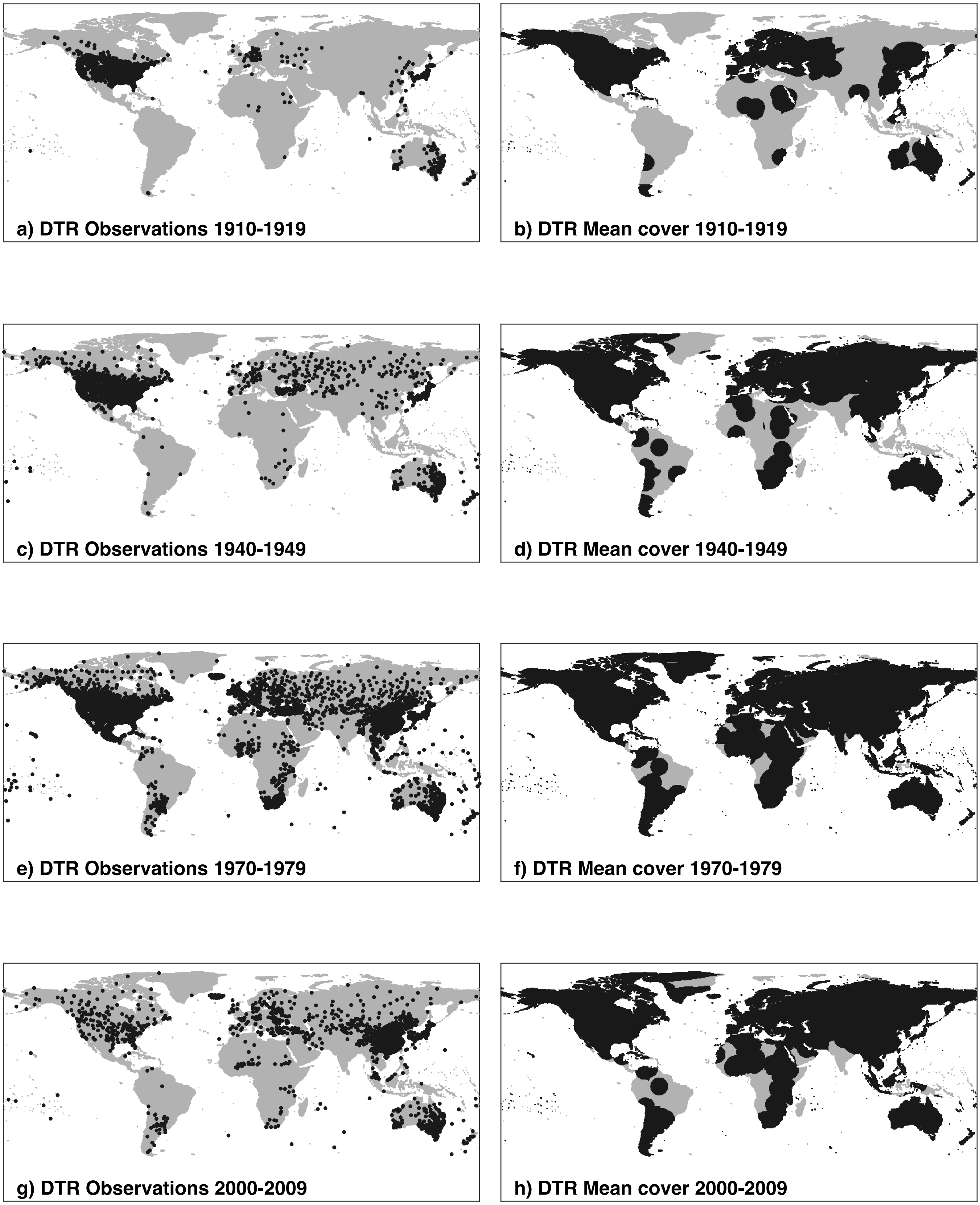

Station locations and coverage for DTR (diurnal temperature range) for four decades

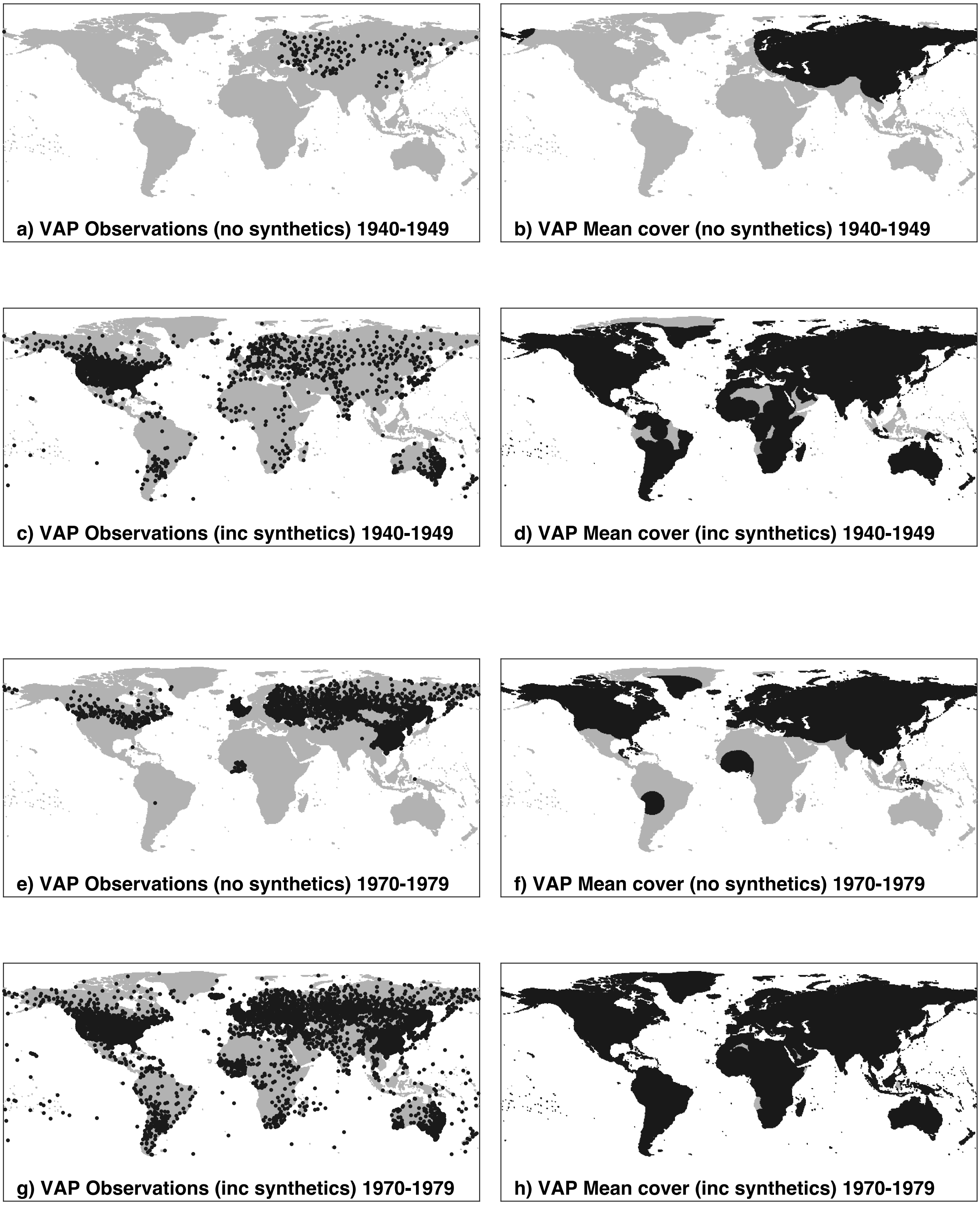

Station locations and coverage for VAP (mean vapour pressure) for two decades, with and without synthetic cover
